# Antiproliferative effect of D-glucuronyl C5-epimerase in human breast cancer cells

**DOI:** 10.1186/1475-2867-10-27

**Published:** 2010-08-19

**Authors:** Tatiana Y Prudnikova, Liudmila A Mostovich, Natalia V Domanitskaya, Tatiana V Pavlova, Vladimir I Kashuba, Eugene R Zabarovsky, Elvira V Grigorieva

**Affiliations:** 1 Institute of Molecular Biology and Biophysics SD RAMS, Timakova str., 2, Novosibirsk, 630117 Russia; 2 MTC, Department of Clinical Science and Education, Sodersjukhuset, Karolinska Institute, Nobels vag 16, Stockholm, 17177 Sweden; 3 Institute of Molecular Biology and Genetics, UNAS Zabolotnogo str., 150, Kiev, 03680 Ukraine; 4 Engelhard Institute of Molecular Biology RAS, Vavilova str., 32, Moscow, 119991, Russia

## Abstract

**Background:**

D-glucuronyl C5-epimerase (GLCE) is one of the key enzymes in the biosynthesis of heparansulfate proteoglycans. Down-regulation of *GLCE *expression in human breast tumours suggests a possible involvement of the gene in carcinogenesis. In this study, an effect of *GLCE *ectopic expression on cell proliferation and viability of breast carcinoma cells MCF7 *in vitro *and its potential molecular mechanisms were investigated.

**Results:**

*D-glucuronyl C5-epimerase *expression was significantly decreased in MCF7 cells compared to normal human breast tissue. Re-expression of *GLCE *inhibited proliferative activity of MCF7 cells according to CyQUANT NF Cell Proliferation Assay, while it did not affect their viability in Colony Formation Test. According to Cancer PathFinder RT Profiler PCR Array, antiproliferative effect of *GLCE *in *vitro *could be related to the enhanced expression of tumour suppressor genes р53 (+3.3 fold), E2F1 (+3.00 fold), BRCA1 (+3.5 fold), SYK (+8.1 fold) and apoptosis-related genes BCL2 (+4.2 fold) and NFKB1 (+2.6 fold). Also, *GLCE *re-expression in MCF7 cells considerably changed the expression of some genes involved in angiogenesis (IL8, +4.6 fold; IFNB1, +3.9 fold; TNF, +4.6 fold and TGFB1, -5.7 fold) and invasion/metastasis (SYK, +8.1 fold; NME1, +3.96 fold; S100A4, -4.6 fold).

**Conclusions:**

The ability of *D-glucuronyl С5-epimerase *to suppress proliferation of breast cancer cells MCF7 through the attenuated expression of different key genes involved in cell cycle regulation, angiogenesis and metastasis molecular pathways supports the idea on the involvement of the gene in regulation of breast cancer cell proliferation.

## Background

D-glucuronyl C5-epimerase (GLCE) is one of the key enzymes responsible for biosynthesis of the carbohydrate part of heparan sulfate proteoglycans (HSPGs) - complex protein-carbohydrate molecules localized on the cell surface and in extracellular matrix (ECM). HSPGs interact with numerous ligands, including many growth factors, cytokines, receptors and extracellular matrix molecules and mediate cell signaling events controlling migration, proliferation and differentiation [1-4]. Abnormal expression or deregulated function of these proteoglycans crucially affect cell-cell and cell-matrix interactions and promote different pathologies including malignant transformation [5,6].

In many cases, the structure of the heparan sulfate (HS) polysaccharide chains is a major determinant of HSPGs function [7]. Changes in expression of heparan sulfates, as well as of enzymes involved in their biosynthesis and degradation, contribute to different steps of tumour progression [8] and study of the heparan sulfate biosynthesis system has a critical importance since its defect affects all HSPGs synthesised by the cell [5].

One of the key enzymes of HS biosynthesis is D-glucuronyl C5-epimerase that is responsible for epimerization of D-glucuronyl residue (D-GlcUA) into L-iduronyl residue (L-IdoUA) in HS carbohydrate chains [9]. Up to date, there are not so many data concerning mammalian *D-glucuronyl C5-epimerase *and no data on human *GLCE*. The gene was cloned from bovine lung [10], mouse liver [11] and mouse mastocytoma cells [12]; knockdown of a murine glucuronyl C5-epimerase gene resulted in neonatal lethality of experimental animals [13]. It was shown that the gene is involved in the embryonic development of Danio rerio [14] and its expression is regulated via beta-catenin-TCF4 transactivation pathway [15].

Some indirect data also support an importance of *GLCE *in cell physiology - a presence of flexible IdoUA residues in HS is necessary for the interaction of heparan sulfates with FGF2 and subsequent cell signaling [16] and for the interaction of hepatocyte growth factor/scatter factor with its signaling receptor MET [17].

Our previous data on significant down-regulation of *GLCE *expression in human breast tumours suggested a possible involvement of the gene in carcinogenesis [18,19].

We hypothesized that *GLCE *expression could be involved in regulation of breast cancer cell proliferation through the changed structure/composition of cell surface heparan sulfates and tumour microenvironment. To test this hypothesis, we ectopically expressed D-glucuronyl C5-epimerase in MCF7 breast cancer cells at the physiological level and analyzed a proliferative activity and viability of the epimerase-expressing cells as well as possible molecular mechanisms of the functional effect of *GLCE in vitro*.

## Results

### *D-glucuronyl C5-epimerase *cloning

To study a functional role of *D-glucuronyl C5-epimerase *in human breast cancer cells, it was necessary to have the gene cloned into the specific plasmid vector for the effective expression in mammalian cells. As a human *D-glucuronyl C5-epimerase *has not been cloned, we amplified a full-length *GLCE *from the KIAA-00836-pBluescript plasmid (Kazusa DNA Research Institute, Japan) containing 5'-truncated epimerase fragment. A long 59 DNA oligonucleotide was used as a forward primer to re-build a 5'-missing part of the gene. Amplified full-length DNA fragment was cloned into episomal vector рСЕР4 (Invitrogen). Nucleotide sequence of *D-glucuronyl C5-epimerase *was verified by sequencing and the obtained DNA plasmid was designated as epi-pCEP4.

### *D-glucuronyl C5-epimerase *expression in human breast cancer cells MCF7

**Figure 1:**
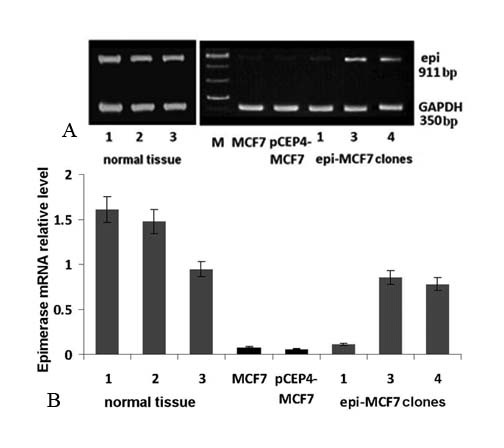
***D-glucuronyl C5 epimerase *expression in normal human breast tissue and breast cancer cells**. A - multiplex RT-PCR with primers for *GLCE *and *GAPDH*; B - *GLCE *expression referenced to GAPDH (TotalLab Рrogramme, Nonlinear Dynamics, UK). The data are shown as mean ± S.D.

Previously, it was shown that *D-glucuronyl C5-epimerase *expression is significantly down-regulated in 82-84% of the human breast tumours [19]. As a first step of the study, *GLCE *expression in human breast cancer cells MCF7 was estimated using multiplex RT-PCR (Fig.1).

According to our results, *D-glucuronyl C5-epimerase *expression is significantly decreased in MCF7 cells compared with the normal human breast tissue. Transfection of the cells with epi-pCEP4 plasmid restored a *GLCE *expression almost up to physiological level in epi-MCF7 clones 3 and 4. Epi-MCF7 clone 1 did not expressed *GLCE *in spite of plasmid presence and was considered as a control as well as MCF7 cells transfected with an empty vector pCEP4 (pCEP4-MCF7 cells).

### An influence of *D-glucuronyl C5-epimerase *on viability and proliferation of breast carcinoma cells MCF7 *in vitro*

**Figure 2:**
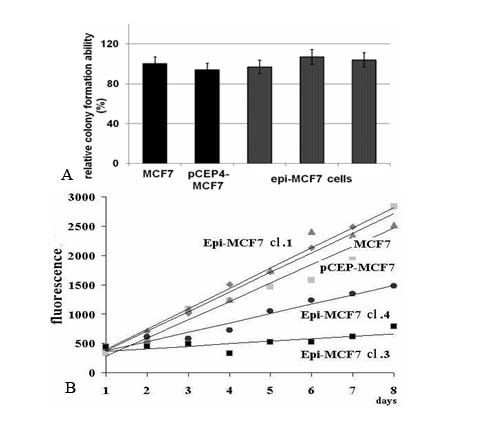
**Effect of *D-glucuronyl C5-epimerase *on viability and proliferation rate of human breast cancer cells**. Colony formation efficiency (A) and proliferative activity (B) of epimerase-expressing breast cancer cells MCF7. pCEP4-MCF7 - control MCF7 cells transfected with рСЕР4 vector; 1,3,4 - epi-MCF7 clones.

Viability and proliferation rate of breast cancer cells *in vitro *were investigated using Colony Formation Test and the CyQUANT NF Cell Proliferation Assay (Invitrogen) (Fig.2).

It was demonstrated that *D-glucuronyl C5-epimerase *ectopic expression did not influence the viability of breast cancer cells MCF7 in Colony Formation Test - the number of epi-MCF7 colonies per 10 mm plate was even higher than those on the plates with MCF7 and pCEP4-MCF7 cells (Fig.2A). However, a difference in growth rate of the epi-MCF7 and pCEP4-MCF7 colonies suggested a different proliferation rate of the cells.

Proliferative activity of the cells stably expressing D-glucuronyl C5-epimerase (epi-MCF7 clones 3 and 4) and control MCF7 cells (pCEP4-MCF7 and epi-MCF7clone 1) was determined using the CyQUANT NF Cell Proliferation Assay. The assay is based on measurement of cellular DNA content via fluorescence dye binding and designed to produce a linear analytical response from at least 100-20,000 cells per well in most cell lines in a 96-well microplate. Fluorescence intensities were measured every 24 hours and plotted on diagram (Fig.2B). According to this assay, *D-glucuronyl C5-epimerase *re-expression in epi-MCF7 cells (clones 3 and 4) significantly (4-5 fold) suppressed their proliferative activity compared with the control cells, not expressing epimerase (MCF7, pCEP4-MCF7 and epi-MCF7 clone 1).

Therefore, re-expression of *D-glucuronyl C5-epimerase *in human breast cancer cells MCF7 significantly suppressed proliferative activity of the cells without affecting their viability, suggesting regulatory rather than suppressing function of the gene.

### Molecular mechanisms of antiproliferative effect of *D-glucuronyl C5-epimerase*

**Figure 3:**
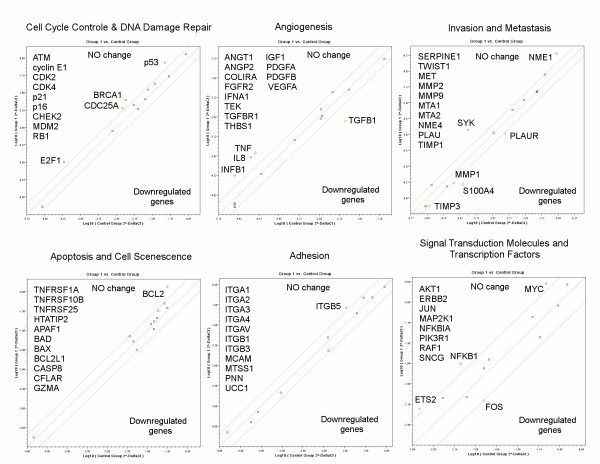
**Cancer PathFinder RT2 Profiler™ PCR Array analysis of epimerase-expressing breast cancer cells MCF7**. The relative expression levels for each gene in the epimerase-expressing MCF7 cells (Group 1) are plotted against the ones from the control pCEP4-MCF7 cells (Control Group) in the Scatter Plot. Diagonal shows the same expression in both groups with 2-fold change boundaries. Genes up-regulated in the epi-MCF7 cells more than 2-fold are marked above the middle lane and down-regulated genes are marked below the lane.

To reveal possible molecular mechanisms of the antiproliferative effect of *D-glucuronyl C5-epimerase *to human breast cancer cells *in vitro*, Cancer PathFinder RT Profiler PCR Array (SABiosciences) was used. This system profiles the expression of 84 genes representative of the six biological pathways involved in carcinogenesis (cell cycle control, signal transduction, apoptosis and cell senescence, angiogenesis, adhesion, invasion and metastasis) (http://www.sabiosciences.com/rt_pcr_product/HTML/PAHS-033A.html). The relative expression levels of each gene in any two samples (experimental and control) are plotted against each other in the Scatter Plot revealing genes up- and down-regulated in the experimental sample. In this study, epimerase-expressing MCF7 cells (clones 3 and 4) were compared with the control pCEP4-MCF7 cells (Fig.3).

**Figure 4:**
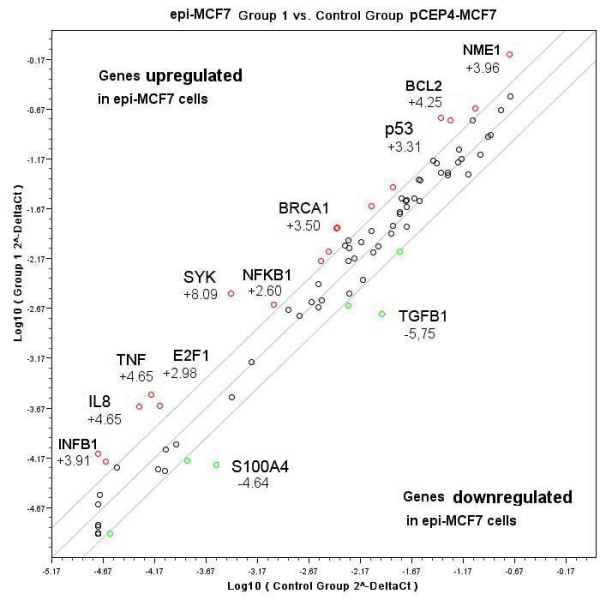
**Up- and Down-regulated genes in *D-glucuronyl C5-epimerase*-expressing breast cancer cells MCF7**. The relative expression levels for each gene in epi-MCF7 (Group 1) and pCEP4-MCF7 (Control Group) cells are plotted against each other. Diagonal marks an equal expression of the gene, bounded region corresponds to 2-fold change of expression.

It was shown that expression of 22 genes was either down- or up-regulated more than 2 fold while the expression of the remaining 62 genes were unaffected. To analyze the combination and functional importance of the influenced genes, we placed all genes with more than 2-fold expression change in epi-MCF7 cells in one diagram (Fig.4).

Re-expression of *D-glucuronyl C5-epimerase *in breast cancer cells MCF7 influenced the expression of some cancer-related genes involved mostly in three molecular pathways:

1. cell cycle control and DNA damage repair (up-regulation of tumour-suppressing genes р53 (+3.3 fold), BRCA1 (+3.5 fold), SYK (+8.1 fold) and transcription factor E2F1 (+3.00 fold));

2. angiogenesis (changes in the expression of IL8 (+4.6 fold), IFNB1 (+3.9 fold), TNF (+4.6 fold) and TGFB1 (-5.7 fold));

3. invasion and metastasis (up-regulation of metastasis-suppressor genes SYK (+8.1 fold) and NME1 (+3.96 fold) and down-regulation of metastasis associated gene S100A4 (-4.6 fold)).

Also, up-regulation of apoptosis-related genes BCL2 (+4.2 fold) and NFKB1 (+2.6 fold) was detected.

The obtained data indicated that antimitotic effect of *D-glucuronyl C5-epimerase *in human breast cancer cells *in vitro *probably is realized mainly via the activation of tumour suppressor genes р53, BRCA1, SYK and E2F1 and change of a balance of pro- and anti-apoptotic factors BCL2, NFKB1 and TNF.

## Discussion

The primary aim of this study was to investigate a possible interrelation between *D-glucuronyl C5-epimerase *expression and proliferative activity of human breast cancer cells. The obtained results support the hypothesis that *GLCE *re-expression in human breast cancer cells MCF7 indeed suppresses their proliferation *in vitro. *It is not something unexpected - tumour-suppressor function was shown for another HS-synthesizing enzyme EXT1, which is responsible for the initial step of heparan sulfate (HS) chain elongation [20]. It was shown that epigenetic inactivation of *EXT1*, leading to an abrogation of HS biosynthesis, is common for leukemia and non-melanoma skin cancer, and reintroduction of EXT1 into HS-deficient leukemia cells HL-60 suppressed colony formation and growth of tumour xenografts [20]. *Ext1 *mutation in mouse embryonic fibroblasts resulted in substantially reduced HS chains length, reduced ability to attach to collagen I and impaired FGF2 signaling that also could be rescued by reintroduction of *Ext1 *[21]. In our target cell line MCF7, *EXT1 *is actively expressed and HS chains are present in the cells [20] but *GLCE *expression is almost abrogated. It suggests a decreased IdoUA content in heparan sulfate chains that is critically important for interaction of the HS with growth factors and appropriate cell signaling [16,17]. The changes in HS structure could influence, in turn, a gene expression profile of the cells and various molecular mechanisms involved in the regulation of cell proliferation.

Thus, our results suggest *D-glucuronyl C5-epimerase *as a second candidate tumour-suppressor gene (TSG) from the HS biosynthetic machinery, whose inactivation in breast cancer cells MCF7 could contribute to breast tissue-specific malignant transformation.

To reveal the molecular mechanisms of antiproliferative effect of *D-glucuronyl C5-epimerase *in breast cancer cells MCF7, PathFinder RT Profiler PCR Array was applied. Analysis of the obtained results showed that molecular mechanisms of cell cycle control, angiogenesis and invasion/metastasis were affected in the epimerase-expressing MCF7 cells.

In our *in vitro *experimental system, re-expression of *D-glucuronyl C5-epimerase *in breast cancer cells MCF7 significantly (3-8 fold) increased expression level of the breast tumour suppressor genes BRCA1 [22] and SYK (Spleen Tyrosine Kinase) [23] which are known as important inhibitors of breast cancer cell growth and metastasis. Other cancer-related genes influenced by the ectopic expression of *GLCE *were transcription factors p53 and E2F1 that are key molecules of the INK4A-Rb-E2f and ARF-MDM2-p53 pathways and pivotal regulators of cell proliferation and viability. Recent data reveal that there is extensive crosstalk between the pathways and in particular between the transcription factors E2F1 and p53 which cooperate in inhibition of tumourigenesis by activation of many pro-apoptotic genes and inducing cell death [24]. From the other side, E2F-containing protein complexes mediate p53-induced growth arrest and senescence indicating the contradictory role of E2F1 in tumourigenesis that is context dependent and tissue specific [24].

In this study, the ectopic expression of human *D-glucuronyl C5-epimerase *in MCF7 cells increased the expression of both p53 and E2F1 but the expression of almost all tested apoptosis-involved genes were unaffected and viability of the epimerase-expressing MCF7 cells were even slightly higher that the control ones (Fig.3). It suggests that GLCE-activated p53 and E2F1 expression more likely affects molecular pathways promoting growth arrest and senescence rather than apoptotic functions of both p53 and E2F1, like their potentially crucial regulator SIRT1 [24]. The hypothesis is also supported by an increased expression of the anti-apoptotic genes BCL2 [25], TNF and transcriptional factor NF-kappaB which protects breast cancer cells from apoptosis [26].

Taken together, these data suggest that up-regulation of the expression of the tumour-suppressor genes (р53, BRCA1, SYK) and transcription factor E2F1 could be responsible for the antimitotic effect of *D-glucuronyl C5-epimerase *in human breast cancer cells *in vitro.*

Another important observation was that ectopic D-glucuronyl C5-epimerase expression led to significant change of the expression of genes involved in angiogenesis (IL8, IFNB1, TNF) and invasion/metastasis (SYK, NME1, S100A4, TGFβ). Among them, there are both microenvironment-dependent regulators of tumour initiation, progression and metastasis like TGFβ that has diverse and often conflicting roles in tumour progression [27] and tumour- and metastasis-suppressor gene SYK [23], metastasis suppressor gene NME1 [28] and metastasis-associated gene S100A4 (MTS1, metastasin) [29].

Interestingly, all those changes have a clearly anti-metastatic trend - re-expression of *GLCE *in breast cancer cells MCF7 up-regulates the expression of suppressor genes SYK and NME1 and down-regulates the expression of metastasis marker S1004A and TGFβ. It is not something completely unexpected since the process of metastasis is dependent on adhesion properties of cells and their interaction with tumour microenvironment, which are determined in significant extent by the structure and composition of heparan sulfates at ECM and cell surface. From the obtained data, we can hypothesize that the expression of D-glucuronyl C5-epimerase in breast cancer cells not only inhibits their proliferation *in vitro *but could suppress tumour growth and metastasis *in vivo *through the influence on cell microenvironment and tumour angiogenesis, which could be one of the future perspectives to study.

## Conclusions

In summary, the obtained results showed that *D-glucuronyl C5-epimerase *significantly suppress proliferative activity of breast cancer cells *in vitro *through the activation of tumour suppressor genes р53, BRCA1, SYK and E2F1 and change of a balance of pro- and apoptotic factors BCL2, NFKB1 and TNF. The ability of *D-glucuronyl C5-epimerase *to affect simultaneously several different key genes of the cell cycle regulation (р53, E2F1, BRCA1), angiogenesis (IL8, IFNB1, TNF, TGFB1) and metastasis (SYK, NME1, S100A4, TGFb) supports the idea on the involvement of the gene in regulation of breast cancer cell proliferation.

## Methods

### Cell lines and culture conditions

Human breast cancer cell line MCF7 was obtained from Karolinska Institute (Stockholm, Sweden). Cells were maintained in IMDM medium supplemented with 2 mM L-glutamine, 100 units penicillin 100 μg/ml streptomycin and 10% (v/v) fetal bovine serum at 37°C in a humidified 5% CO_2_. For analysis cells were harvested by standard protocol using trypsin/EDTA (Sigma, USA).

### Human *D-glucuronyl C5-epimerase *cloning

Human *D-glucuronyl C5-epimerase *(*GLCE*) (NM_015554) was cloned into episomal vector pCEP4 (Invitrogen, USA). Full-length sequence of epimerase cDNA was amplified by PCR with *Pfu*-DNA-polymerase (Stratagene) and epi-oligo-F 5'-CTAAGATCTAGATATGCGTTGCTTGGCAGCTCGGGTCAACTATAAGACTTTGATTATTA-3' and epi-R 5'-TACAGCGGCCGCTGAAGTGCAGTTTTGGT-3' primers from KIAA0836 clone (AB_020643) coding 5'-truncated sequence of the *GLCE *(Kazusa DNA Research Institute, Japan). Amplified full-length fragment was cloned into pCEP4 vector and obtained plasmid was defined as epi-pCEP4. *GLCE *sequence was verified by sequencing (Dye Terminator Cycle sequencing Kit and ABI PRISM 3700 genetic analyzer).

### Transfection and selection of stable cell clones

To obtain stable cell clones expressing *D-glucuronyl C5-epimerase*, MCF7 cells were transfected with epi-pCEP4 or pCEP4 plasmid DNA (0.5 μg DNA/well) using Lipofectamine-ReagentPlus combination (Invitrogen, USA) according to the manufacturer's protocol. Stable cell clones were selected for 2-3 weeks in IMDM medium containing 200 μg/ml Hygromycin (Sigma, USA).

### Colony formation assay

pCEP4- or epi-pCEP4-transfected MCF7 cells were stripped 24-48 h after transfection and plated on 100 mm cell culture dishes at 500-1000 cells per plate. After selection by 200 μg/ml Hygromycin for 2 weeks, giemsa-stained colonies were photographed and counted by Quantitione software, Version 4.4.0 (Bio-Rad, USA).

### RT-PCR analysis of *GLCE *expression

Total RNA was isolated from the cells using PureLink Total RNA Purification System (Invitrogen, Carlsbad, CA) according to manufacturer's protocol. Synthesis of cDNA was performed from 1 μg of total mRNA using First Strand cDNA Synthesis kit (Fermentas, Hanover, MD) according to manufacturer's instruction. Multiplex PCR was performed in 20 μl volume (200 ng cDNA, 2 μl 10*PCR buffer 10 mM Tris-HCl, 1,5 mM MgCl2, 50 mM KCl, pH 8,3, 5 pmole of each primer, 0,2 mmole of each dNTP and 1 unit Taq-Polymerase) in Tercik PCR machine (DNA Technology, Russia). PCR conditions: 95°C 10 min, 95°C 15 s, 59°C 15 s and 72°C 1 min with final elongation at 72°C 10 min; 32 cycles for *GAPDH *and 20 cycles for *GLCE*. Following primers were used:

*GLCE*-F, 5'-AAGGGAGACGAGAGGGGAACGAA-3'; *GLCE*-R, 5'GCCACCTTTCTCATCCTGGTTC-3'; GAPDH-F, 5'-GGGCGCCTGGTCACAA-3'; GAPDH-R, 5'-AACATGGGGGCATCAGCAGA-3'.

Products of amplification were analyzed by electrophoresis in 1.2% agarose gel and visualized using "DNA Analyzer" system (Moscow, Russia). *GLCE *expression level was estimated as a ratio of *GLCE *DNA fragment intensity to *GAPDH *DNA fragment intensity (TotalLab Рrogramme, Nonlinear Dynamics, UK).

### Cell proliferation assay *in vitro*

Cell proliferation rate was estimated using the CY QUANT NF Cell Proliferation Assay (Invitrogen, USA) according to manufacturer's instruction. Cells were plated at densities 100-500 cells/well to 8-12 wells in a 96-well microplate. DNA content in the wells was measured every 24 hours - the medium was removed and 50 μl fluorescent dye was added followed by incubation for 30 min at 37°C. Fluorescent intensity of each sample was measured at 485/530 nm with Fluorescence Microplate Reader (SPECTRA max, "Molecular Devices", UK).

### Cancer-related genes expression profiling

To identify possible molecular mechanisms of antiproliferative effect of *GLCE*, Cancer PathFinder RT2 Profiler™ PCR Array (SABioscience, Frederick, MD) was used. Total RNA was isolated using RNAqueous Micro Kit (Applied Biosystems, Foster City, CA) according to manufacturer's protocol. RNA concentration was measured with Quant-iT Assay Kit and Qubit instrument (Invitrogen, USA), RNA quality was evaluated by electrophoresis. cDNA was synthesized from 1-2 μg total RNA using First Strand cDNA Synthesis kit (Fermentas, Hanover, MD). Real-time PCR was performed with SYBR Green PCR Master Mix (Fermentas, Hanover, MD) and iCycler iQ5 Multicolor Detection System (Bio-Rad, USA) according to manufacturer's instructions. Obtained data were analyzed with Excel-based PCR Array Data Analysis Software (SABioscience, Frederick, MD).

## Competing interests

The authors declare that they have no competing interests.

## Authors' contributions

TYP carried out most of the experiments, LAM carried out the research and drafted the manuscript, NVD carried out the molecular genetic studies, TVP carried out the tissue culture work, VIK participated in coordination of the study, ERZ participated in coordination of the study and contributed to the writing of the manuscript, EVG designed the experiments and wrote the manuscript. All authors have read and approved the final manuscript.
